# Brain tumor classification: a novel approach integrating GLCM, LBP and composite features

**DOI:** 10.3389/fonc.2023.1248452

**Published:** 2024-01-30

**Authors:** G. Dheepak, Anita Christaline J., D. Vaishali

**Affiliations:** Department of Electronics & Communication Engineering, Faculty of Engineering and Technology, SRM Institute of Science and Technology, Vadapalani Campus, Chennai, TN, India

**Keywords:** brain tumor, GLCM, LBP, texture, composite feature, aggregated feature, non-linear feature

## Abstract

Identifying and classifying tumors are critical in-patient care and treatment planning within the medical domain. Nevertheless, the conventional approach of manually examining tumor images is characterized by its lengthy duration and subjective nature. In response to this challenge, a novel method is proposed that integrates the capabilities of Gray-Level Co-Occurrence Matrix (GLCM) features and Local Binary Pattern (LBP) features to conduct a quantitative analysis of tumor images (Glioma, Meningioma, Pituitary Tumor). The key contribution of this study pertains to the development of interaction features, which are obtained through the outer product of the GLCM and LBP feature vectors. The utilization of this approach greatly enhances the discriminative capability of the extracted features. Furthermore, the methodology incorporates aggregated, statistical, and non-linear features in addition to the interaction features. The GLCM feature vectors are utilized to compute these values, encompassing a range of statistical characteristics and effectively modifying the feature space. The effectiveness of this methodology has been demonstrated on image datasets that include tumors. Integrating GLCM (Gray-Level Co-occurrence Matrix) and LBP (Local Binary Patterns) features offers a comprehensive representation of texture characteristics, enhancing tumor detection and classification precision. The introduced interaction features, a distinctive element of this methodology, provide enhanced discriminative capability, resulting in improved performance. Incorporating aggregated, statistical, and non-linear features enables a more precise representation of crucial tumor image characteristics. When utilized with a linear support vector machine classifier, the approach showcases a better accuracy rate of 99.84%, highlighting its efficacy and promising prospects. The proposed improvement in feature extraction techniques for brain tumor classification has the potential to enhance the precision of medical image processing significantly. The methodology exhibits substantial potential in facilitating clinicians to provide more accurate diagnoses and treatments for brain tumors in forthcoming times.

## 1 Introduction

A tumor is an abnormal growth of cells which occurs in any portion of the human body. Over two hundred various kinds of cancer, such as lung, blood, breast, heart, lymphoma, etc. have been reported (1). According to World Health Organization (WHO) fact sheet 2022, cancer has been the leading cause of death with 10 million deaths reported (2). Among the various types of tumors, brain tumors have been the primary reason for death in various age and gender groups and are also challenging to treat. A tumor in the human brain is a collection of malignant cells which develops when brain tissues suddenly and abnormally extend. There are different types of brain tumors, some are non-cancerous (benign) and some are cancerous (3). The human brain acts as the body’s control hub. It coordinates the actions of vast numbers of neurons and their many connections. Tumor in the brain disrupts normal brain activities and the nervous system processes. The need to overcome the disadvantages of manual tumor image analysis, which is both time-consuming and vulnerable to human subjectivity, motivated machine learning based techniques of classifying tumors.

As discussed by Abdusalomov et al. (4) Glioma, Meningioma, Pitutary seem to be the common types of brain tumors that look like non-cancerous, but may be. Hence this research intends to study these brain tumors and classify them by incorporating advanced features such as GLCM and LBP, as well as interaction features and statistical analysis. This method has the potential to significantly improve the precision of medical image processing for more precise brain tumor identification and treatment planning.

The primary contributions of this present investigation are

- This research introduces a novel methodology for comprehensive texture analysis of tumor images. The approach integrates Gray-Level Co-occurrence Matrix (GLCM) and Local Binary Pattern (LBP) features. GLCM features capture spatial pixel intensity relationships, while LBP features identify local texture patterns. Together, these features provide a detailed insight into tumor textures, facilitating improved understanding and tumor classification.
- A novel feature generation technique has been proposed, which generates interaction features by multiplying GLCM and LBP feature vectors. This technique generates a new set of features that enhance the discriminative ability of the model, thereby enhancing its capacity to differentiate between various tumor morphologies. The incorporation of these interaction features enables the acquisition of a broader spectrum of texture data, resulting in a greater comprehension of tumor characteristics.
- In addition, the methodology incorporates the computation of aggregated characteristics derived from GLCM properties. These aggregated features, which consist of the sum, mean, and median of the GLCM features, provide a more comprehensive view of the overall characteristics of tumor textures.
- For Tumor classification Support Vector Machine (SVM) is implemented with extracted features which enhances the performance of tumor classification.

The next section discusses the various research works related to tumors and their classification.

## 2 Related literature

In order to categorize common brain tumor types, Kaplan et al. (5) have used nLBP and αLBP feature extraction approaches. Using the K-Nearest Neighbour (Knn) model and the nLBPd = 1 method, they achieved a high 95.56% success rate of identifying tumors. Another study by Abdusalomov et al. (4) used YOLOv7 and transfer learning to improve brain tumor diagnosis in MRI scans, they report an outstanding 99.5% accuracy for identifying the most common types of brain tumors Glioma, Meningioma, Pitutary. However, they also acknowledge the need for additional research, particularly for minor tumor identification (4).

Research by Kaya et al. (6) used a novel feature extraction technique based on co-occurrence matrices from vibration data to address the problem of accurate bearing issue identification. Effective success rates were obtained by utilizing 1D-LBP and machine learning: 87.50% for dataset 1 (various speeds), 96.5% for dataset 2 (fault size in mm), and 99.30% for dataset 3 (fault type - inner ring, outer ring, ball). Study by Solani et al. (7) examines the difficulties in diagnosing brain tumors and provides information on the potential of MR imaging. They adopt statistical and machine learning techniques to detect brain tumor for a chosen dataset. Yildirim et al. (8) have studied the most accepted forms of brain tumors include gliomas, meningiomas, and pituitary. They say that the optimal course of action for treating these tumors may differ reliant on the type. Brain tumors can be challenging to classify, even for experts, due to heterogeneous imaging findings (8).

According to Shinde et al. (9), while progress has been made in classifying anomalies in medical imaging, there are still challenges to overcome. These include, but are not limited to, model selection, data description, error detection, data sufficiency, and result reliability. As a result, there is no one highest benchmark for categorizing medical images. So, it is quite problematic in computer vision and machine learning domains. The algorithms mentioned generally are developed using soft computing and model-based methodologies, and their results are reliable (9). With the help of mobile sensor inputs, work by Kuncan et al. (10) presents a unique feature extraction approach called DS-1D-LBP for human activity recognition (HAR). They have successfully classified with the Extreme Learning Machine (ELM) with a high success rate of 96.87%. Research by Shil et al. (11) rely heavily on features that have been manually constructed and then provided to a classifier, such as Support Vector Machine, Decision Tree, or k-Nearest Neighbour (KNN). Khalid et al. state that the extensive nature of the dataset can cause delays in feature engineering, thereby increasing the likelihood of errors and highlighting the significance of domain expertise (12).

Processing and analyzing MRI images of brain tumors is one of the most challenging and promising new areas of study. An MRI, which employs magnetic fields and radio waves to generate overall images of internal body structures, is essential for determining the optimal course of treatment for a tumor and its progression. Texture features based on GLCM were first introduced by Haralick et al. (13) in 1979. In biomedical field, advantage of the textural properties of images aids in image classification.

There are numerous methods available to derive the relevant data from imaging modalities for region-based segmentation, including artificial neural network (ANN), fuzzy clustering means (FCM), support vector machine (SVM), knowledge-based techniques, and the expectation-maximization (EM) algorithm technique. Image analysis using SVM and BWT methods was suggested by Bahadure et al. (14) for detecting and classifying brain tumors using MRI. Skull stripping, in which non-brain tissues are removed, allowed for a 95% detection rate utilizing this method. Joseph et al. (15) introduced a technique for segmenting MRI brain images for tumor diagnosis that combines the K-means clustering algorithm and morphological filtering technique. Alfonse and Salem (16) suggested a method for automatically classifying MRI scans for brain tumors using a support vector machine. The researchers employed Fast Fourier Transform (FFT) to extract features to enhance the classifier’s precision. Additionally, they utilized technology that exhibited minimal redundancy and maximum relevance to reduce the number of features.

In order to better segregate brain tumors, Shree et al. (17) pre-processed the images using multiple noise removal methods. Their research employed DWT and GLCM-based characteristics of brain tumors. Any residual noise after segmentation was filtered out using morphological filtering procedures. The suggested model was trained and evaluated using the probabilistic neural network classifier for pinpointing tumor locations in brain MRI scans. Yao et al. (18) provided a method that includes extracting texture characteristics using the wavelet transform and classifier as SVM with an accuracy of 83% to process and address protocols of diverse images and non-linearity of actual data to classify improved MRI images related to contrast. Principal component analysis (PCA) and a radial basis function kernel with SVM were proposed by Kumar and Vijayakumar (19) for classifying and segmenting brain tumors. They were able to achieve 94% success rate using this strategy.

Saleck et al. (20) developed a Fuzzy C-Mean (FCM) way of figuring out the size of a patient’s brain tumor. They could figure out how many groups were there in the FCM by looking at the intensity of each pixel. This approach uses GLCM texture feature extraction to forecast the threshold value. The generic performance of a model is established by how sensitive, specific, and accurate it is.

Considering the research going on in this field, it seems evident that choosing appropriate features of tumor images and adopting appropriate machine learning classifiers will lead to better identification of tumors.

Based on the literature survey, this research intends to study three types of brain tumors (Glioma, Meningioma, Pitutary) and classify them by incorporating novel and advanced features such as GLCM and LBP, as well as interaction features and statistical analysis. This research work introduces novel contributions in the following aspects:

- **Interaction Features:** The extraction of interaction features involves the computation of the outer product between the Gray-Level Co-occurrence Matrix (GLCM) and Local Binary Pattern (LBP) feature vectors. The aforementioned process results in the formation of a matrix that effectively encompasses the interplay between spatial and local texture data. The matrix is subsequently converted into a unidimensional array in order to constitute the collection of interaction features.
- **Non-linear Features:** Another significant contribution is applying a logarithmic transformation to the GLCM features. This transformation generates non-linear features, allowing for the capturing of complex relationships within tumor images. By incorporating these non-linear features, the research improves the discriminative power of the feature set and enables a more sophisticated analysis and interpretation of the tumor image properties.

## 3 Proposed methodology

**Figure 1:**
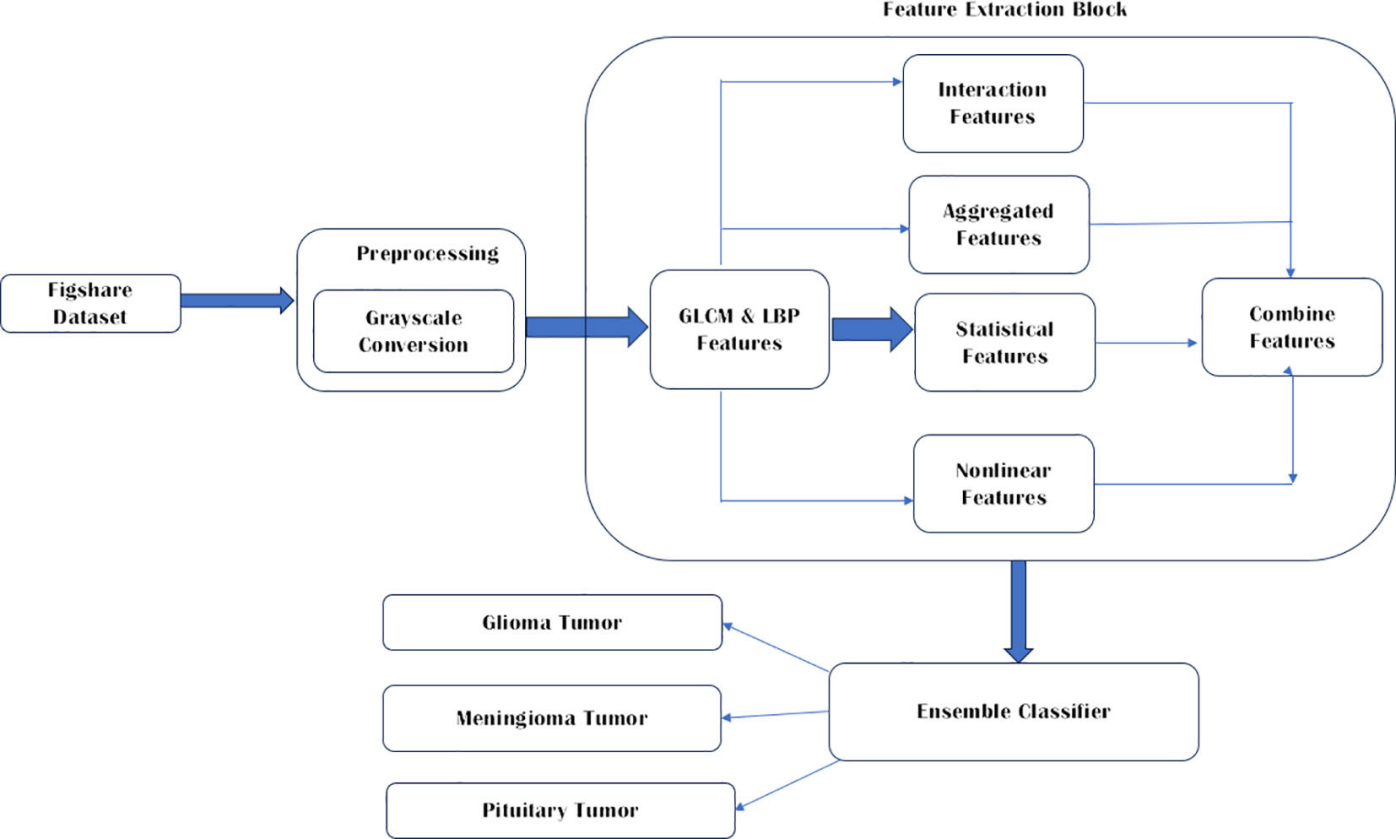
Overall proposed model architecture.

Publicly available databases Figshare Dataset (13) has been used in this study since it is one of the most common datasets used by many other researchers. This brain tumor dataset contains 3064 T1-weighted contrast enhanced images from 233 patients for three kinds of brain tumor: meningioma (708 slices), glioma (1426 slices), and pituitary tumor (930 slices) (21). The conversion to grayscale from RGB is performed to enrich the images further. The three brain tumor images used are Glioma, Meningioma, and pituitary tumor. As proposed by Demirhan et al. (22) converting the input images to grayscale gives a simplified representation is obtained that effectively captures the overall brightness information while eliminating the complexities associated with color. The parameters include boosting the signal-to-noise ratio, making MR images look better, removing the background of unwanted sections, smoothing the inner parts and keeping the essential edges intact. The proposed work is depicted in **Figure 1**.

## 4 Feature extraction

To extract significant features from brain MRI images, six types of feature extraction techniques have been implemented in this study, as listed in **Table 1**. Using these techniques, important aspects of the images could be identified and analyzed.

**Table 1:** Feature etxtraction Techniques used in this research work.

S.No	Feature Extraction Technique used
1	Grey Level Co-occurrence Matrix (GLCM) Features
2	Local Binary Patterns (LBP) Features
3	Interaction Features
4	Aggregated Features
5	Statistical Features
6	Nonlinear Features

### 4.1 GLCM feature extraction

Texture analysis facilitates the differentiation between healthy and unhealthy tissues for visual perception and ML algorithm. In addition, it reveals differences between malignant tumors and normal tissues that might not be observable by the naked eye. By selecting efficient statistical features for early diagnosis, the accuracy can be improved. Second-order statistical texture features can be extracted using GLCM. Creating a GLCM matrix and then deriving statistical metrics from this matrix measures the frequency with which pairs of pixels with specific values and a specific spatial relationship appear in an image. GLCM, or Gray-level spatial dependence matrix (GLSDM), has been used in this research used to extract the statistical features. GLCM was first proposed by Haralick et al. (23) for describing the geographical relationship between pixels with different levels of Gray-level.

To analyze an image’s texture statistically, the GLCM counts how often pairs of pixels with the same value and the same relative position appear in the image. By determining the frequency with which pairs of pixels with a given weight and in a given spatial relationship occur in an image, the GLCM functions can characterize an image’s texture through the extraction of statistical measurements. The Gray-Level Co-occurrence Matrix (GLCM) is a two-dimensional histogram where each pair of ‘p’ and ‘q’ represents the frequency with which the events ‘p’ and ‘q’ occur. As a function of distance S = 1, angle (0 degrees horizontal, 45 degrees positive diagonal, 90 degrees vertical, and 135 degrees negative diagonal), and gray scales ‘p’ and ‘q’, it determines the frequency with which a pixel of intensity ‘p’ occurs in proximity to a pixel of intensity ‘q’ at a given distance ‘S’ and orientation.

After computing GLCM, five different statistical features are extracted from GLCM. These extracted features include,

**Contrast:** Determines the local variances of the grey-level co-occurrence matrix.

**Homogeneity**: Determines the proximity of the GLCM element distribution to the GLCM diagonal.

**Dissimilarity:** Quantifies the range of grayscale intensity.

**Energy**: It will calculate the pixel’s uniformity.

**Correlation**: Calculates the average degree to which each pixel in the image is connected with its neighbors.

The formulas used to calculate the above characteristic features are shown in **Table 2**.

**Table 2:** Features of GLCM.

**Contrast**	$\sum^{}{(i-j}^{2})\;*\;(P\;i,j)$
**Dissimilarity**	$=\sum^{}P_{i,j}\left|i-j\right|$
**Homogeneity**	$=\sum^{}\frac{P\left(i,j\right)}{\left(1+\left|i-j\right|\right)}$
**Energy**	$=\sum^{}\frac{P\left(i,j\right)}{\left(1+\left|i-j\right|\right)}$
**Correlation**	$=\sum^{}\frac{\left(i-\mu i\right)\left(j-\mu J\right)}{\left(\sigma i*\sigma j\right)}$

### 4.2 LBP features

The Local Binary Patterns (LBP) technique is a widely employed texture descriptor in image processing and computer vision. Local texture representation is a straightforward yet efficient method used to depict the texture characteristics of an image. This technique has been extensively applied in various domains, such as object recognition, face detection, and image segmentation. The step wise LBP Feature Extraction is, each pixel is compared with its neighboring pixels. Consider the Gray value of the center pixel as *g_c_*, and the gray value of the neighboring pixel as *g_p_*. Comparison is carried out with using Equation 1.


(1) $$S\left({ɡ}_{p},{ɡ}_{c}\right)=if\;{ɡ}_{p}\geq ={ɡ}_{c}\;else\;0$$

A circle of radius R is centered around the center pixel and the function S is applied to P evenly spaced pixels.

### 4.3 Interaction features

A novel approach implemented in this research is the extraction of image features for tumor classification, wherein a strategy for generating interaction features is employed with the aim of potentially improving the performance of the model. This approach entails the integration of two sets of important attributes: the GLCM attributes and LBP attributes, both of which effectively capture fundamental properties of the images being analyzed.

The GLCM features provides insights into the spatial interdependence of pixel intensities within an image, effectively capturing and representing texture details. In contrast, the features of LBP provide a quantification of the local spatial patterns of pixel luminance, thereby providing supplementary information regarding texture.

The computation of interaction features involves the outer product of the feature vectors obtained from GLCM and LBP. In mathematical terms, the outer product of the GLCM features vector (g) and the LBP features vector (l) yields a matrix (M). Each element *M_i,j_* of this matrix represents the product of the i^th^ GLCM feature and the j^th^ LBP feature. The aforementioned statement aptly describes the correlation between the respective GLCM and LBP features.

Subsequently, the interaction matrix is transformed into a one-dimensional vector, thereby generating a novel set of features that capture the interplay between the initial feature sets. The inclusion of this extended feature set, in combination with the existing GLCM and LBP features, offers a more exhaustive and refined depiction of the image. Consequently, it has the potential to improve the machine learning model’s capacity to differentiate between various tumor classifications.

The steps involved in interaction features are,

**Defining GLCM and LBP features vector:** GLCM feature vector is defined as

*ɡ = ɡ_1_, ɡ_2_, ɡ_3_,…ɡ_m_* and the LBP feature vectors as *l = l_1_,1_2_,l_3_,…l_n_* in which ‘m’ is the number of GLCM features and ‘n’ is number of LBP features.

**Calculating the outer product:** The matrix ‘M’ is obtained by computing the outer product of the two given vectors. The matrix is provided as follows:


(2) $$M=ɡ⊗\text{l}=\;\left[\begin{array}{ccc} {ɡ}_{1}l_{1} & \cdots & {ɡ}_{1}l_{n}\\ \vdots & \ddots & \vdots \\ {ɡ}_{m}l_{1} & \cdots & {ɡ}_{m}l_{n} \end{array}\right]$$

Each element of this matrix *M_ij_* = *ɡ_i_l_j_* is a new feature created which captures the interaction between *i^th^* GLCM feature as well as with *j^th^* LBP feature.

**Combining interaction features and original features:** The original GLCM and LBP features are combined with the interaction features to create the final feature vector for each image. If the GLCM feature vector ‘g’ has a size of m and the LBP feature vector ‘l’ has a size of n, then the final feature vector will have a size of *m* + *n* + *mXn*.

These procedures describe how interaction features are generated from GLCM and LBP features. This augmented feature set has the potential to provide a more comprehensive and nuanced image representation, thereby enhancing the performance of the image classification model.

Here GLCM calculates at 4 different angles such as (0, 45, 90, and 135 degrees). The GLCM algorithm proceeds by computing five distinct properties, namely ‘contrast’, ‘dissimilarity’, ‘homogeneity’, ‘energy’, and ‘correlation’, for each angle. These properties are then used to generate a vector of GLCM features. Assuming the vector representing the GLCM features for an image is [10, 20, 30, 40, 50]. The aforementioned values indicate that the contrast is 10, the dissimilarity is 20, the homogeneity is 30, the energy is 40, and the correlation is 50.

The LBP algorithm is employed to calculate the LBP values using 8 sampling points positioned evenly along a circle with a radius of 1. Subsequently, the histogram of these LBP patterns is computed. Suppose the Local Binary Patterns (LBP) features for a given image are represented by a histogram consisting of 256 bins. The values within this histogram range from 0.01 to 0.01, with each bin containing a distinct value.

The interaction features are generated through the computation of the outer product between the feature vectors of the Grey Level Co-occurrence Matrix (GLCM) and the Local Binary Patterns (LBP).

For the purpose of explanation, consider a simplified scenario where only first five bins of LBP features. These bins are represented by the values [0.01, 0.02, 0.03, 0.04, 0.05].

The 5x5 matrix is obtained by computing the outer product of the GLCM features [10, 20, 30, 40, 50] and the first 5 LBP features [0.01, 0.02, 0.03, 0.04, 0.05]. The value of each element in this matrix is obtained by multiplying a feature from the Grey Level Co-occurrence Matrix (GLCM) with a feature from the Local Binary Patterns (LBP) feature.

The 5x5 matrix is obtained by taking the outer product of the GLCM features [10, 20, 30, 40, 50] and the first 5 LBP features [0.01, 0.02, 0.03, 0.04, 0.05]. The value of each element in this matrix is obtained by multiplying a feature derived from GLCM with a feature derived from the LBP algorithm as represented in Equation 3.


(3) $$\left[\begin{array}{ccc} 10*0.01 & \cdots & 10*0.05\\ \vdots & \ddots & \vdots \\ 50*0.01 & \cdots & 50*0.05 \end{array}\right]$$

The interaction features offer way to capture the potentially significant connections among various elements of an image’s texture. The precise dynamics of these interactions are dependent upon the data and the attributes of the images under examination. In the context of tumor images, these interactions may potentially expose complicated patterns that are pivotal in distinguishing between various tumor types.

### 4.4 Aggregated features

The aggregated features represent the additional statistical features of GLCM features. The GLCM features can be consolidated into more straightforward statistical measures. The mean, median, and total of the GLCM characteristics are these statistical measurements. The steps involved in aggregated features calculation is shown in **Algorithm 1**.

**Algorithm 1:** Calculation of aggregated features.

1: **Input**: GLCM Features 2: **Output**: Aggregated Features 3: Initialize sum as 0 4: **for** each GLCM feature in the input do 5: Add the GLCM feature to the sum 6: **end for** 7: Compute mean as sum divided by the total number of GLCM features 8: Sort the GLCM features in ascending order 9: Compute the median based on the sorted list of GLCM features 10: **Output** the sum, mean, and median as the aggregated features

The features extracted from aggregated features are as follows,

**Sum:** The total number of GLCM characteristics is determined. This gives us a single number that can be taken as a measure of the “amount” of GLCM features in the image. If the GLCM characteristics are represented by the vector g = [*ɡ*1, *ɡ*2,…*ɡn*] then

*ɡ_m* = *ɡ*1 + *ɡ*2 + … + *ɡn* gives the total.

**Mean:** The average GLCM characteristics are determined. This gives us a single number that stands in for the “typical” value of the GLCM feature in the image.

$G_{Mean}=\frac{\left(ɡ1+ɡ2+\ldots ɡn\right)}{n}$ gives the mean, where ‘n’ defines the GLCM features total numbers.

**Median:** GLCM features median are computed. When the GLCM features are ordered numerically, this yields a single value that characterizes the “middle” value. The median is the midpoint if ‘n’ is an odd number. If ‘n’ is divisible by 2, then the median is the midpoint between those two values.

Following these computations, the aggregated GLCM features take the form of a vector with three elements, which are denoted by the notations [*ɡ_sum, ɡ_mean, and ɡ_median*].

The aggregated features (sum, mean, and median) provide a summary of the GLCM features, capturing various aspects of their level and distribution. In addition to the GLCM features, LBP features, interaction features, statistical features, and non-linear features, these characteristics are added to the final feature vector for each image.

Pattern recognition, machine learning, and image classification employ aggregated features such as summation, average, and median. The raw features are condensed, effectively representing the central tendency and overall pattern of the data, thereby offering a simplified yet meaningful perspective of the dataset. The aforementioned properties exhibit a lower degree of variation compared to individual data points, thereby enhancing the robustness of models against the presence of noisy or outlier data. The utilization of these techniques results in a reduction of data dimensionality, thereby enhancing the efficiency of algorithms. The inclusion of aggregated features in a model has been observed to enhance its predictive performance by uncovering latent data patterns. The code provided utilizes the GLCM attributes to generate aggregated features that summarize the textural characteristics of the image. This has the potential to enhance the tumor classification model.

### 4.5 Statistical GLCM features

A set of statistical measures derived from GLCM of an image constitutes the statistical GLCM features. The GLCM matrix depicts the spatial relationship among image pixel pairs. Each element of the GLCM indicates the probability that two pixels with a particular grey level will occur at a particular distance apart. GLCM statistical traits are used to describe an image’s texture (24). Texture is how the pixels in an image are placed in space. It can be used to tell the difference between different kinds of images, like images of nature, medical images, and images of factories, etc.

The following features are derived under statistical GLCM features,

**Variance:** The variance quantifies the degree to which GLCM features deviate from their mean value. When the variance of the GLCM features is high, the values they take on span a wide range, whereas when it’s low, the features tend to cluster tightly around the mean. Equation 2 represent the variance formulation. Provided that GLCM feature vector *ɡ* = [*ɡ*_1_, *ɡ*_2_…*ɡ_m_*]. Where ‘m’ is the number of GLCM features.


(4) $$variance=\;=\;\frac{1}{m}\sum_{i=1}^{m}{\left({ɡ}_{i}-mean\right)}^{2}$$

Where, average of GLCM features is mean.

**Skewness:** The concept of skewness pertains to the degree of asymmetry exhibited by the distribution of GLCM features in relation to their mean value. Skewness is computed as Equation 5,


(5) $$skewness=\;\frac{1}{m}\sum_{i=1}^{m}{\left(\frac{{ɡ}_{i}-mean}{std}\right)}^{3}$$

Where ‘std’ is the standard deviation of GLCM features.

**Kurtosis:** Kurtosis assesses the tailedness of GLCM feature distributions. High kurtosis shows thick tails and a strong peak, indicating many outliers. Low kurtosis shows light tails and a flat peak, indicating no outliers. Mathematically Kurtosis is expressed in Equation 6.


(6) $$kurtosis=\;\frac{1}{m}\sum_{i=1}^{m}{\left(\frac{{ɡ}_{i}-mean}{std}\right)}^{4}-3$$

From Equations 3, 4 ‘m’ is the number of GLCM features and *ɡ_i_* refers to the i^th^ element of GLCM feature vector.

These statistical GLCM characteristics can shed light on how the GLCM features are typically distributed. Statistical GLCM features exhibit information on the texture’s variability, asymmetry, and outliers, while GLCM features themselves capture the texture details within the image.

The incorporation of statistical GLCM features, such as variance, skewness, and kurtosis, enhances the predictive model by encompassing supplementary distributional information pertaining to the GLCM features. The statistical measures employed in this study shed light on subtle texture variations that may not be easily distinguishable solely from the raw GLCM features, as they effectively capture the spread, asymmetry, and tailedness of these features. The model’s exceptional performance metrics, such as its nearly perfect accuracy, precision, recall, and F1 score, are likely enhanced by the incorporation of statistical GLCM features, although other factors may also contribute to these outcomes. The inclusion of these features enhances the diversity of the 1547-feature set, thereby augmenting the model’s capacity to accurately classify the tumor images. Statistical GLCM features play a crucial role in enhancing the predictive performance of the model by providing detailed information regarding the texture characteristics of the image.

### 4.6 Non-linear features

Another set of novel features of this research work, in terms of non-linear features, is computed from the GLCM feature vectors by applying a logarithmic transformation. This process generates non-linear features from the GLCM feature vectors. These GLCM feature vectors, obtained from grayscale image analysis, contain numerical values representing various statistical measures. The application of the logarithmic transformation on these GLCM feature vectors gives rise to the non-linear features. These non-linear features capture intricate patterns and relationships in the data, which may enhance the performance of machine learning models.

The procedure for obtaining non-linear features from the features derived from the Grey Level Co-occurrence Matrix (GLCM) is as follows,

**GLCM feature computation:** The initial stage entails the computation of the Gray-Level Co-occurrence Matrix (GLCM) features, which serve to extract texture information from the image. The aforementioned features consist of contrast, dissimilarity, homogeneity, energy, and correlation. The computations pertain to the manipulation of the Grey Level Co-Occurrence Matrix (GLCM), a matrix that denotes the occurrence frequency of various combinations of pixel intensities within the image.

The GLCM features can be denoted as C(contrast), D(dissimilarity), H(homogeneity),

E (energy), and Corr(correlation).

**Applying Non-linear Transformation:** Once the GLCM features have been computed, a non-linear transformation is applied to these features by utilizing the natural logarithm function. The transformation is represented by,


(7) nq.logq(m)=log(m+1)

From Equation 7 where, ‘m’ as the scalar value or the array of the input for which the natural log is to be determined and the input array is being incremented by ‘1’ which is referred as ‘m+1’ in Equation 3. Later, the modified array log function is ‘(m+1)’ which is being computed for each element present in GLCM vector. The resultant of this log function is the non-linear features.

The computation of the non-linear features corresponding to each GLCM feature is performed in the following manner.


(8) $$C^{\prime }=\text{log }\left(1+C\right)$$

(9) $$D^{\prime }=\text{log }\left(1+D\right)$$

(10) $$H^{\prime }=\text{log }\left(1+H\right)$$

(11) $$E^{\prime }=\text{log }\left(1+E\right)$$

(12) $$Corr^{'}=\text{log }\left(1+Corr\right)$$

Where Equations 8-12 represent the transformed GLCM features.

In the given transformation, it is crucial to add one to the original feature value, denoted as C, before applying the natural logarithm function, represented as C’ = log(1+C). The need for this adjustment arises in cases where the correlation coefficient ‘C’ is equal to zero, as the natural logarithm of zero is undefined. This lack of definition can result in computational challenges. The inclusion of a constant term enables the logarithmic transformation to be applicable to all conceivable values of ‘C’, encompassing the value of zero. This step is crucial in ensuring the integrity and precision of the feature engineering process. The identical process is employed for the remaining GLCM features, ensuring the integrity of all modified features.

The primary objective of employing the logarithmic transformation is to effectively capture and accentuate non-linear relationships and variations present within the GLCM features. Through the utilization of the logarithm function, the feature values undergo a transformation, resulting in their representation on a logarithmic scale. The utilization of this technique can facilitate the identification of patterns, intensify the differentiation between elements, and enhance the accuracy of the representation of the characteristics of the GLCM.

Non-linear transformations have the potential to effectively capture complex structures within the data, which may not be easy to identify through the original features. Consequently, the utilization of such transformations has the capacity to enhance the performance of machine learning models.

### 4.7 Concatenated features

Finally, the various features including GLCM, LBP, interaction, aggregated, statistical, and non-linear features are consolidated into a unified feature vector for every image. The process involves arranging all the features consecutively to create a lengthy vector. The concatenated feature vector serves as a representation of the image within the feature space, enabling its utilization in subsequent analysis or machine learning endeavours.

## 5 Results & discussion

The brain tumor classification model was implemented in Python. Statistical and non-linear feature extraction were used in conjunction with GLCM, LBP, Interaction, and aggregation to create the model. Finally, we classified brain tumors using a support vector machine.

**Proposed Composite Feature Extraction Model Performance**

In this work, a set of performance metrics have been computed to evaluate the effectiveness of the proposed composite Feature Extraction model, as illustrated in **Table 3**.

**Table 3:** Performance metrics formula.

Metric	Formula
Accuracy	(TP + TN)/(TP + TN + FP + FN)
Precision	TP/(TP + FP)
Recall	TP/(TP + FN)
F1-score	2 * (precision * recall)/(precision + recall)
Sensitivity	TPR
Specificity	TNR
TPR	TP/(TP + FN)
FPR	FP/(FP + TN)
FNR	FN/(TP + FN)
TNR	TN/(FP + TN)

**Tables 4**–**8** compares the proposed Composite Feature Extraction model’s sensitivity, precision, specificity, accuracy, DSC, FPR, and FNR metrics with 23 existing models.

**Table 4:** Comparison of performance metrics: existing models with composite feature extraction (proposed model).

Author/Name of Model	Precision	Recall or sensitivity	F1-score	Specificity	Accuracy
Gupta et al, 2019 (24)	98.84	97.25	97.21	98.12	96.28
Rasool et al (25),	98.1	98.00	–	–	98.12
Fine-tuned EfficientNetB2, 2023 (26)	98.65	98.77	–	99.34	98.86
DAWE Model, 2021 (27)	97.4	95.6	–	96.9	99.3
**proposed Composite Feature Extraction Model**	**99.837**	**99.836**	**99.836**	**99.836**	**99.83**

The bold letter used are highlighting the proposed model used in the manuscript.

**Table 5:** DSC comparison: composite feature extraction vs. existing models.

Name of the Model	Dice Similarity Coefficient (DSC)
HOG + LBP + deep features, 2021 (28)	96.11
RG + MKM + U-NET, 2020 (29)	90
DAWE Model, 2021 (27)	96.5
Multiscale Cascaded Multitask Network, 2023 (30)	96.21
**Proposed Composite Feature Extraction Model**	**99.6**

The bold letter used are highlighting the proposed model used in the manuscript.

**Table 4** presents a comprehensive evaluation of performance metrics for different models, including the Composite Feature Extraction model proposed in this study. The assessed metrics include Precision, Recall (or sensitivity), F1-score, Specificity, and Accuracy.

The proposed Composite Feature Extraction model demonstrates exceptional performance in brain tumor classification, achieving approximately 99.83% across all key metrics: Precision, Recall, F1-score, Specificity, and Accuracy. The obtained result indicates the model’s high reliability in accurately identifying tumors and healthy cases, effectively reducing false outcomes. The balanced F1-score shows the model’s consistent performance in precision and recall. Overall, the model’s robustness and superior performance signify its effectiveness in brain tumor classification, surpassing existing models.

The presented **Table 5** provides a comparative analysis of the Dice Similarity Coefficient (DSC) values of different models utilized to classify three distinct types of brain tumors, namely glioma, meningioma, and pituitary tumors. The Dice similarity coefficient (DSC) is an essential metric to quantify the degree of similarity between the predicted and actual tumor regions observed in brain images. A greater DSC (Dice similarity coefficient) value indicates a higher level of accuracy in the model, particularly in accurately classifying and distinguishing glioma, meningioma, and pituitary tumors. As proposed, the Composite Feature Extraction model demonstrates exceptional performance with a Dice Similarity Coefficient (DSC) value of 99.6. This value signifies the model’s superior accuracy in effectively classifying the three distinct types of brain tumors, surpassing the performance of existing models.

**Table 6** illustrates the Composite Feature Extraction model’s enhanced efficacy in classifying tumors. The model demonstrates superior performance compared to existing models, as evidenced by its remarkably low False Positive Rate (FPR) of 0.00625 and a False Negative Rate (FNR) of 0.0. These results highlight the model’s exceptional accuracy and reliability in effectively reducing false alarms and missed detections.

**Table 6:** FPR and FNR comparison: composite feature extraction vs. existing models.

Model	FPR	FNR
DAE-JOA, 2020 (31)	0.46	0.04
Stacked auto-encoder, 2019 (32)	0.07	0.1
DWAE model (27)	0.0625	0.031
**Proposed Composite Feature Extraction Model**	**0.00625**	**0.0**

The bold letter used are highlighting the proposed model used in the manuscript.

**Table 7** presents the categorization of Meningioma, Glioma, and Pituitary Tumours utilizing various machine-learning techniques. The combined studies used a total of 3064 samples. The proposed methodologies for image classification include a Convolutional Neural Network (CNN) model achieving an accuracy of 97.3%. Additionally, a hybrid approach combining Convolutional Dictionary Learning and AlexNet achieves a 91-96% accuracy range. Another model, BrainMRNet, incorporates hypercolumns, attention modules, and residual blocks, achieving an accuracy range of 96-98%. The proposed Composite Feature Extraction model utilizing GLCM, LBP, and Composite Features achieves an accuracy of 99.83% compared with other existing models.

**Table 7:** Comparison based on dataset: composite feature extraction vs. existing models.

Author	Brain Tumor classes	Image Dataset	Feature Extraction/selection	Accuracy
Fransisco Javier Diaz-pernas, MPDI 2021	Meningioma, Glioma, and Pituitary Tumor	3064	CNN model	97.3
XiaoqingGu, Neuroscience, 2021	Meningioma, Glioma, and Pituitary Tumor	3064	Convolutional dictionary learning+AlexNet	91-96
Mesut T, Springer 2021	Meningioma, Glioma, and Pituitary Tumor	3064	BrainMRNet, including hypercolumn technique, attention modules, and residual blocks	96-98
**Proposed Composite Feature Extraction model**	**Meningioma, Glioma, and Pituitary Tumor**	**3064**	**GLCM, LBP, and Composite Features**	**99.83**

The bold letter used are highlighting the proposed model used in the manuscript.

In this research work, SVM classifier with a linear kernel is implemented to classify different types of tumors. The input data is processed and converted into the desired format using the kernel function. The complexity of a linear Support Vector Machine (SVM) is lower than that of a non-linear SVM, resulting in faster training.

Support Vector Machines (SVMs) can effectively classify data points by projecting them onto a feature space with many dimensions, even in cases where the data points are not linearly separable. Once a separator between the categories has been identified, the data is transformed, representing the division as a hyperplane. In the present context, the performance metrics of accuracy, precision, recall, and F1 score indicate that SVM can effectively discriminate between various classes of brain tumors. Accurately classifying brain tumors is paramount in medical diagnosis and subsequent treatment planning.

The comparison of various techniques for classifying brain tumors is presented in **Table 8**. Several models are included in this study, such as BMRI-NET, which is a stack ensemble model. Additionally, a two-channel deep neural network (DNN) model, GoogleNet with K-nearest neighbors (KNN), VGG-16, Resnet50, and InceptionV3 models, a simple convolutional neural network (CNN), a multiscale cascaded multitask network, and the DL (ResNet50V2) model are also considered. The accuracy of the data falls within the range of 96.3% to 99.68%.

**Table 8:** Accuracy comparison: composite feature extraction vs. existing models.

Author	Technique used	Accuracy (%)
Asif et al., 2023 (33)	BMRI-NET (Stack ensemble model)	98.69
Jyostna et al., 2021 (34)	Two-channel DNN model	98.04
Deepak and Amir 2019 (35)	GoogleNet + KNN	98.00
Alshayeji et al., 2021 (36)	Concatenation of 2 CNN	97.37
Patel M 2023 (37)	VGG-16, Resnet50, InceptionV3	0.975 for VGG-16, 0.95 for Resnet50, 0. 915 for InceptionV3
Latif G (2022) (38)	CNN	96.30
Z. Sobhaninia et al. (2023) (30),	Multiscale Cascaded Multitask Network	97.98
Md.A. Talukder et al. (2023) (39),	DL (ResNet50V2)	99.68
**Proposed Composite Feature Extraction model**	**GLCM, LBP, and Composite Features+SVM-Linear**	**99.83**

The bold letter used are highlighting the proposed model used in the manuscript.

The highest accuracy of 99.83% was achieved by employing a combination of Composite Feature Extraction techniques, namely Grey Level Co-occurrence Matrix (GLCM), Local Binary Patterns (LBP), and Composite Features, in conjunction with an SVM-Linear classifier. The findings of this study indicate that the technique examined in this research is the most prominent method for classifying brain tumors when compared to other approaches, exhibiting exceptional levels of accuracy.

**Figure 2:**
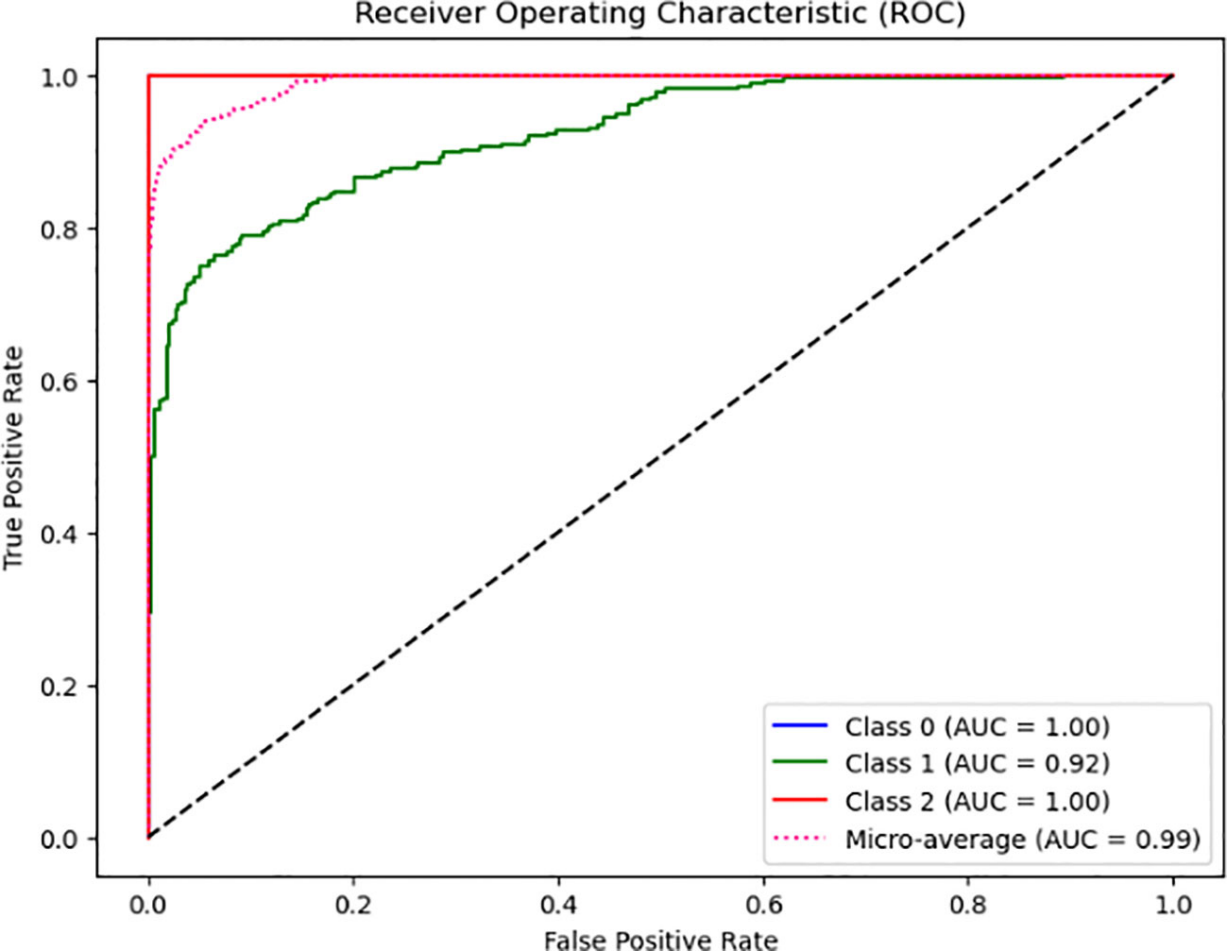
Proposed composite feature extraction model ROC plot.

From **Figure 2**, the model’s classification performance is considered exceptional based on the Area Under the Curve (AUC) values obtained from the Receiver Operating Characteristic (ROC) analysis. The model achieves a perfect Area Under the Curve (AUC) score of 1.00, indicating its ability to classify instances belonging to Class 0 and Class 2 accurately. Despite encountering challenges and exhibiting a few inaccuracies, the model’s overall performance remains commendable, evidenced by its high Area Under the Curve (AUC) value of 0.92. However, it is essential to consider the comprehensive evaluation of the model’s performance. It is noteworthy that the micro-average AUC achieves a near-perfect score of 0.99, indicating the model’s high efficacy across all classes.

A significant amount of time, specifically 654.45 seconds, was dedicated to the analysis of image features, such as the Grey Level Co-occurrence Matrix (GLCM) and Local Binary Pattern (LBP), during the extraction process. However, the training process of the Support Vector Machine (SVM) classifier using these features was completed in a mere 0.16 seconds. The utilization of resources in this study was primarily focused on feature extraction rather than model training. With a strikingly low ratio of 0.00024389, the outstanding efficiency of computation is evident in this scenario, clearly demonstrating the high priority of computing resources.

The model attained a perfect average accuracy score of 1.0 on the training data, regardless of the varying sizes of the training sets. Nevertheless, notwithstanding the flawless score, the model exhibited commendable performance on the validation data. The predictive capabilities of the model exhibit a high level of robustness, as indicated by a mean accuracy score that falls within the range of 0.993 to 0.998. Furthermore, it is worth noting that the model consistently demonstrated strong performance across a diverse set of data folds, as indicated by the narrow range of standard deviations, which varied between 0.013 and 0.035.

## 6 Conclusion

Based on the obtained results, the classification model demonstrates exceptional performance, with an accuracy, precision, recall, and F1 score that are all close to 1, indicating a high success rate in classifying across all three classes of brain tumors (Glioma, Meningioma, Pitutary). The model also demonstrates a 100% True Positive Rate (no false negatives) and a 0% False Negative Rate (no false positives), in addition to a very low False Positive Rate, indicating an excellent balance between sensitivity and specificity.

This work presents a novel methodology that offers an improved representation of tumor image textures by combining LBP and GLCM features. The addition of interaction features, which are created by taking the outer product of the GLCM and LBP vectors, greatly improves the derived features’ ability to discriminate. This unique feature distinguishes the strategy from other approaches. Furthermore, an even more thorough representation of critical tumor image characteristics is obtained through the integration of aggregated, statistical, and non-linear information. Combining the approach with a linear support vector machine classifier yields 99.84% of accuracy rate. We opted to use the Figshare dataset so as to compare our classification accuracies with existing research done with same dataset. Being better than other research outcomes, this method can be applied to real time data.

As the most time-consuming phase, future research could concentrate on streamlining the extraction of features. In addition, future research may investigate the possibility of utilizing the extracted detailed features with less computational time. Another objective of future research is to analyze and classify high-grade brain tumors comprehensively utilizing high-grade brain tumors dataset to improve an understanding of complex tumors.

## Data availability statement

The original contributions presented in the study are included in the article/supplementary material. Further inquiries can be directed to the corresponding author.

## Ethics statement

Ethical review and approval was not required for the study on human participants in accordance with the local legislation and institutional requirements. Written informed consent from the patients/participants or patients/participants’ legal guardian/next of kin was not required to participate in this study in accordance with the national legislation and the institutional requirements.

## Author contributions

All authors listed have made a substantial, direct, and intellectual contribution to the work, and approved it for publication.
